# Hypogammaglobulinemia in pediatric kidney transplant recipients

**DOI:** 10.1007/s00467-022-05757-1

**Published:** 2022-09-30

**Authors:** Victoria Dimitriades, Lavjay Butani

**Affiliations:** 1 Division of Pediatric Allergy, Immunology and Rheumatology, Department of Pediatrics, University of California, Davis, Sacramento, CA USA; 2 Division of Pediatric Nephrology, Department of Pediatrics, University of California, Davis, 2516 Stockton Blvd, Room 348, Sacramento, CA 95817 USA

**Keywords:** Pediatric transplant, Kidney, Immunoglobulin, Infection

## Abstract

Infections remain the most common cause of hospitalization after kidney transplantation, contributing to significant post-transplant morbidity and mortality. There is a growing body of literature that suggests that immunoglobulins may have a significant protective role against post-transplant infections, although the literature remains sparse, inconsistent, and not well publicized among pediatric nephrologists. Of great concern are data indicating a high prevalence of immunoglobulin abnormalities following transplantation and a possible link between these abnormalities and poorer outcomes. Our educational review focuses on the epidemiology and risk factors for the development of immunoglobulin abnormalities after kidney transplantation, the outcomes in patients with low immunoglobulin levels, and studies evaluating possible interventions to correct these immunoglobulin abnormalities.

## Infections after kidney transplantation

As a consequence of the advances in immunosuppressive management of pediatric kidney transplant (Tx) recipients, the incidence of acute rejection has decreased dramatically over the past three decades [1]. However, this has come at a significant cost — a higher burden of infectious complications. In the early post-Tx period, viral and bacterial infections account for about 25% of all hospitalizations in children, and in the most contemporary era infections have become the leading cause of hospitalization after Tx both in the early and late post-Tx periods [1, 2]. As shown in Table 1, post-Tx infections have been shown to follow a somewhat stereotypical pattern [3]. In the first post-Tx month, infections are typically nosocomial infections related to the surgery and hospitalization or rarely are donor derived. Between month 1 and month 6 is when opportunistic infections, such as EBV and CMV, become problematic. After 6 months, the types of infections depend on the kidney function and consequent intensity of immunosuppression needed, with patients falling into three groups. Those with a well-functioning graft are usually on low-dose immunosuppressive therapy and their infections tend to mirror what is seen in the otherwise healthy community. If patients have experienced rejection and have poorer graft function, they often receive more intense maintenance immunosuppression, or have been treated with aggressive anti-rejection medications. Such patients continue to be at a high risk of opportunistic infections. Finally, a third subset of patients in this late period are dealing with chronic or latent infections which were acquired earlier in the post-Tx period.

**Table 1:**
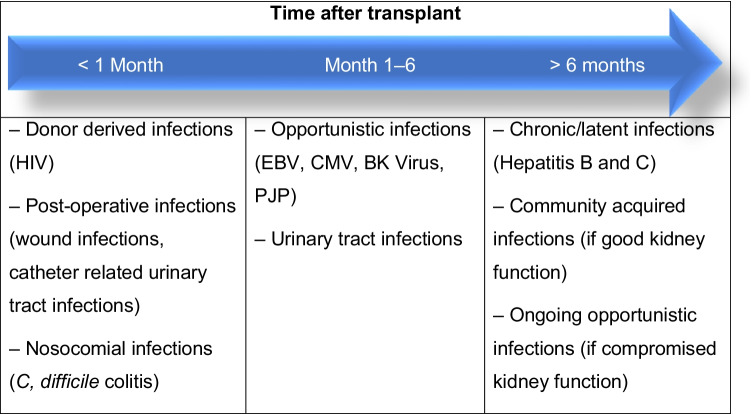
Temporal pattern of infections in the post-transplant period with some examples

Several studies have shown a higher incidence of infections, especially viral infections, and bacterial gastrointestinal infections, in the youngest of Tx recipients, as well as those receiving polyclonal T cell depleting agents [2, 4, 5]. Predisposing factors for urinary tract infections (UTI), another common pediatric post-Tx infection, include the presence of underlying urologic conditions and the use of cyclosporine [6]. In spite of the current approach of screening for infections, pre-emptive therapy of infections and the use of anti-microbial and anti-viral prophylaxis (all of which are applicable only to some infectious organisms) [3], infections remain a major concern after Tx and additional strategies are needed to reduce the morbidity and mortality arising from these. A significant gap in the current post-Tx literature, especially in children, pertains to the epidemiology, risk factors, consequences, and management of patients post-Tx who are noted to have low immunoglobulin (Ig) levels and specific role of Igs as a protective factor in preventing infections.

## Prevalence of hypogammaglobulinemia

Abnormalities in Ig levels have been noted in kidney Tx recipients, both in cross-sectional and in prospective cohort studies. Based on their experience caring for 5 adult kidney Tx recipients who experienced recurrent infections and who were noted to have low IgG levels, Pollock et al. conducted a single-center cross-sectional study of 110 adult renal Tx recipients in 1989 and noted low levels of one or more of the Ig classes in 35% of patients [7]. The only predictor for low Ig levels was a longer duration of immunosuppression.

Since then, the prevalence of Ig abnormalities has been the subject of several large prospective studies, mostly in adult kidney Tx recipients. In 2007, Ig levels were prospectively measured in a cohort of 152 adult kidney Tx recipients, who were receiving calcineurin inhibitors (CNI) like Tacrolimus (Tac), or mycophenolate mofetil (MMF), along with maintenance steroids [8]. Most (82%) had received induction therapy, most commonly with an IL-2 receptor blocker. Secondary hypogammaglobulinemia was defined as an Ig level that was less than the lower limit of normal (normal adult values: IgG: 650–1500 mg/dL; IgA: 75–400 mg/dL; IgM: 40–250 mg/dL). The investigators noted that the proportion of patients with Ig deficiencies increased over time reaching a peak between 1 and 3 months and decreasing by 6–12 months post-Tx: the prevalence of hypogammaglobulinemia was 6% (at baseline), 45% at 3 months and 30% at 12 months. There were no differences either in the prevalence of Ig deficiency, or in the mean Ig levels, when groups who were randomized at 3 months to MMF and steroids were compared to those who were receiving CNI and steroids as dual therapy. No differences were noted in Ig abnormalities when the various induction agents were compared to one another nor with Tac trough levels at 3 months. A small fraction of patients also had multiple Ig abnormalities; at 3 months, 10/150 patients (7%) had deficient IgG and IgA levels, 10/151 patients (7%) had combined IgG and IgM deficiency, and 3/150 (2%) had pan hypogammaglobulinemia.

These findings have been replicated in subsequent prospective studies. In a cohort of 226 adult patients from Spain with a median follow-up of 509 days [9], some of whom were receiving immunosuppression pre-Tx (14%), the prevalence of low IgG increased from 6.6% (baseline) to 52.0% at 1 month and subsequently decreased to 31.4% at 6 months. Of note, 32 patients (20.9%) had persistently low serum IgG levels at both follow-up time points. A similar pattern was noted for other Igs, but at a lower prevalence. Patients were receiving triple immunosuppression with Tac, MMF and steroids and most (80%) had received induction, the majority with anti-thymocyte globulin (48%). The only predictor for low IgG at baseline was the receipt of pre-Tx hemodialysis (compared to continuous ambulatory peritoneal dialysis or no kidney replacement therapy). The presence of low IgG at baseline (odds ratio [OR] 26.9; *p* = 0.012) and a positive anti-HCV status (OR 0.17; *p* = 0.023) in turn emerged as risk factors for the occurrence of post-Tx low IgG.

Similar to previous studies, in one of the largest studies in adult Tx recipients, the prevalence of low IgG was quite high: 56% and 36.8% at post-Tx day 15 and month 6, respectively [10]. Older recipient age was the sole identified risk factor for early hypo-IgG. Risk factors for late hypo-IgG were early hypo-IgG, as shown previously (OR 6.41, *p* < 0.001) and treatment for acute rejection (OR 2.63, *p* = 0.014).

The only prospective pediatric study assessing the prevalence of Ig abnormalities, to our knowledge, evaluated 15 kidney Tx recipients (6 had focal segmental glomerulosclerosis) who had received induction therapy and were on maintenance Tac and MMF, and most were also on steroids [11]. None were receiving immunosuppression pre-Tx. Ig levels in Tx recipients were compared to those in a healthy control group. Mean IgG and IgM levels in this cohort declined to below control levels at 1-month post-Tx; levels increased after that but remained lower than in the control group even at 12–18 months. The prevalence of hypo-IgG was significant and varied from 25% at 1 month to just over 50% at 24 months in this cohort. This study did not separate out and provide detailed information on the subset of pediatric patients who had focal segmental glomerulosclerosis and who may have had significant enough proteinuria to affect Ig levels pre- and post-Tx.

A meta-analysis on the rate of hypogammaglobulinemia in the first post-Tx year among various solid organ Tx recipients [12] confirmed that the overall rate of hypo-IgG (IgG < 700 mg/dL) (16 studies, 1482 patients) was 45% (40% in kidney Tx recipients), the rate of mild hypo-IgG (8 studies, 669 patients) was 39% (46% in kidney Tx recipients), and the rate of severe hypo-IgG (< 400 mg/dL) (8 studies, 669 patients) was 15% (8% in kidney Tx recipients). Children (two studies, 83 patients) had a lower overall rate of hypo-IgG compared to adults (seven studies, 634 patients; 26% vs. 52%; *p* < 0.0001). This meta-analysis included 18 studies, most of which were observational (n 16; 10 retrospective, 6 prospective), with only 2 being prospective randomized trials.

In summary, Ig abnormalities are common in kidney Tx recipients, although they may be somewhat less common in children. A small subset of patients is hypogammaglobulinemic at baseline; the prevalence increases over time with a peak prevalence between 1 and 3 months post-Tx and then decreases. However, a substantial proportion of patients remain hypogammaglobulinemic even as far out as 18–24 months after Tx and a small percentage have multiple Ig abnormalities. Established predictors for post-Tx Ig abnormalities include hypo-Ig at baseline, age at Tx, anti-HCV positivity, and treatment for acute rejection.

## Infection risk and pattern in the setting of Ig abnormalities after transplant

The immune system has many different components, each of which confer protection from different manners of infectious challenge. The innate immune system includes physical barriers (such as the skin and mucosal surfaces), the complement system, and cells such as neutrophils, macrophages, and natural killer cells. When this compartment is compromised, there is an increased rate of infections of those physical barriers, including abscesses, gastrointestinal infections, UTIs, and severe cutaneous viral disease. The adaptive immune system, on the other hand, acts through T cell (cellular immunity) and B cell (humoral, or antibody-mediated immunity) components. With impaired T cell immunity, predominant issues can include severe viral and fungal infections, with an increase in some bacterial infections, as T cells are often dependent on B cell activity. However, B cell impairment, which causes a decrease in antibody production, presents with a much more typical clinical profile. In primary immunodeficiencies, low IgG (which is the predominant antibody in our system) is known to confer a higher risk of bacterial infections from a specific set of encapsulated organisms, such as *Streptococcus pneumoniae*, *Haemophilus influenza*, and *Moraxella catarrhalis* [13]. In general, patients who are hypo-IgG tend to have issues with recurrent otitis, sinusitis, and upper and lower respiratory infections. In a meta-analysis of 676 patients with hypogammaglobulinemia, the trough level of IgG was inversely correlated with the risk of developing pneumonia [14]. In some antibody deficiencies, untreated hypogammaglobulinemia has also been shown to have a direct correlation with increased mortality due to infection as well as contributing to a decreased quality of life [15]. It is also important to note that hypogammaglobulinemia in the setting of primary immunodeficiency is defined at a different level for adults (< 700 mg/dL) than it is for children (in whom age-based reference ranges must be utilized).

Given the knowledge that the adaptive immune system directs antibody-mediated protection (generally sinopulmonary affectation) while the innate immune system directs protection against surface pathogens like those seen in UTIs, it is interesting to note that kidney Tx data shows a much more diverse correlation of infections in the setting of Ig abnormalities, as discussed below.

In the European study by Broeders et al., the influence of Ig abnormalities on infectious complications after Tx was evaluated in 92 adult patients at 3 and 12 months [8]. Overall, UTIs were the most frequent infectious events in this study (cumulative incidence at 12 months 53%), followed by respiratory tract infections (cumulative incidence at 12 months 27%), but these were not statistically different between patients with low or normal IgG at 3 and 12 months post-Tx. What was noted, though, was that the majority of patients with low IgG levels at *baseline* or with *combined* hypogammaglobulinemia [IgG + (IgA and/or IgM)] at 3 months developed infections within the first 3 months, compared to patients free of these abnormalities (*p* < 0.05). The increased risk in the combined group was mainly represented by an increased proportion of patients who developed respiratory infections (43% vs. 5% in patients without combined Ig deficits; risk ratio 9.3 [95% CI 2.6–33.7], *p* = 0.008). In comparison, in the adult cohort from Spain, baseline hypo-IgG was not associated with a higher infection risk in the early post-Tx period [9]. However, patients with hypo-IgG at 1 month exhibited an overall higher incidence of infections between month 1 and 6, specifically bacterial (*p* = 0.004) and fungal infections (*p* = 0.082), blood stream infections (*p* = 0.054), and graft pyelonephritis (*p* = 0.003). The investigators also found a correlation between the severity of hypo-IgG and the incidence of blood stream infections and pyelonephritis; both the incidence rate of overall infection and the mean number of infectious episodes per recipient were also highest in the patients with the most severe IgG deficiency (< 500 mg/dL). Hypo-IgG at 6 months was associated with a higher cumulative incidence of overall infection, bacterial infection, and UTI in the late post-Tx period. Patients with hypogammaglobulinemia of any class also had higher cumulative incidence of late blood stream infection, UTI, and *Clostridium difficile*-associated diarrhea. It must be noted that 35% of the infections noted were UTI/kidney related, 17% were attributed to CMV infections, 12% to bacteremia, and 8% to pneumonia/respiratory tract infections. The bacteria most commonly seen were *Escherichia coli*, *Enterococcus* species, and *Pseudomonas* species. On multivariate regression analysis, the presence of hypogammaglobulinemia of any class at 1 month remained an independent risk factor for bacterial infection in the intermediate post-Tx period (HR 1.81; 95% CI 1.03–3.17). Additionally, hypogammaglobulinemia of any class at 6 months independently predicted the overall occurrence of late infection (HR 2.31; 95% CI 1.18–4.55), as well as late bacterial infection (HR 4.66; 95% CI 1.89–11.48). There was no difference in the overall rate of rejection among groups nor were differences in mean survival noted. However, when only infection-related mortality was assessed, this was significantly higher in the hypogammaglobulinemic patients. Of note, no patients were treated with intravenous Ig (IVIG) [9]. These findings were somewhat in contrast to the study by Augusto et al., where only septicemia occurring between 6 and 12 months post-Tx was more frequently observed in patients with hypo-IgG [10]. However, it is important to note here that this study excluded lower UTIs from their analysis, as they considered those types of infections to be common in all the patients. In comparison to other types of infections, they noted that UTIs were “of little medical significance” as this would not make them more appropriate candidates for IVIG supplementation (which is used, traditionally, to aid in sinopulmonary concerns).

While it seems to be correct that IgG replacement would not necessarily confer better protection towards organisms which do not elicit an adaptive (antibody) immune response, there have been previous studies in other solid organ Tx recipients which show the utility of IVIG in reducing infections from some of these lesser considered organisms. Jordan et al. describe a decrease in the risk of fungal and viral infections in this population (specifically CMV and parvovirus) with monthly dosing of IgG replacement [16]. In a prospective study looking at adult heart transplant patients with pre-Tx hypo-IgG, patients were given monthly IgG and compared to a post-Tx hypo-IgG group which was not being treated. In this clinical trial, patients who received preventive monthly dosing of IgG for 6 months had a lower incidence of CMV disease and Gram-negative infections (due to *E. coli* and *Enterococcus* species) [17]. This same group later looked at infection risk after kidney Tx and found that IgG < 700 mg/dL 1 month after Tx was significantly associated with development of CMV disease and acute pyelonephritis. Infections again tended to center around *E. coli*, *Enterococcus* species, and *Klebsiella* [18].

Finally, in the adult meta-analysis referenced earlier [12], there was an increased risk of infections, in general, linked to hypogammaglobulinemia. In this study, the odds of developing infections in the group with severe hypo-IgG (< 400 mg/dL) was 2.46 times higher than the odds of infection for the group with a serum IgG > 400 mg/dL, while it was even higher when comparing them to the group with IgG > 700 mg/dL (OR 3.73; two studies, 267 patients). When looking at the odds ratios for different types of infections, the severe hypo-IgG group showed a much higher risk than the > 400-mg/dL group in respiratory infections (OR 4.83), CMV infections (OR 2.4), and Aspergillus infections (OR 8.9). The odds of 1-year all-cause mortality in the group with a low IgG level (< 700 mg/dL) was 2.71 times higher than the odds ratio for the group with normal Ig levels (95% CI 1.05–6.99; two studies, 179 patients). Even more striking, the odds ratio for death at 1 year for the severe hypo-IgG group was 21.91 times higher than the odds for the group with IgG > 400 mg/dL (95% CI 2.49–192.55; two studies, 124 patients). In this study, there was no association noted between UTI and Ig abnormalities. However, as the authors mention, the absence of an association between Ig abnormalities and UTIs may be from the small sample; moreover, UTIs are common after kidney Tx, with many potential contributors such as underlying urologic abnormalities (which are common, especially in the youngest of children), bladder catheterization that is universal in the early post-Tx period, Tx vesicoureteral reflux, and the common practice of placing ureteral stents in Tx recipients.

The only pediatric prospective study was small and not designed to effectively address whether Ig abnormalities were associated with a higher risk of infection. Nevertheless, it is worth mentioning that infections were very common in the patients in this study; the most common infections were CMV viremia, UTI, and *Clostridium difficile* infections [11].

The only study, to our knowledge, addressing the impact of IVIG administration in solid-organ Tx recipients with hypogammaglobulinemia was a retrospective investigation of 37 patients (27 were children, 2 of whom had a known primary immunodeficiency) [19]. Hypogammaglobulinemia was diagnosed at median of 5.6 months post-Tx. The 3-year survival after the diagnosis of hypo-IgG was 49.5% (95% CI 32.2–64.6%). Intravenous immunoglobulin products were administered at a median of 2 doses/patient (range 0–38). There was a statistically significant increase in the median IgG levels between the last and first measurements. Patients were dichotomized based upon IgG level at last follow-up: IgG ≥ 400 mg/dL (23 patients) and IgG < 400 mg/dL (14 patients). There was no evidence of a difference in survival (*p* = 0.44), rejection rate (*p* = 0.44), and graft loss censored for death (*p* = 0.99) at 1-year between these two groups. Additionally, there was no difference in survival between patients who were and were not receiving IVIG (*p* = 0.99) or CMV hyperimmunoglobulin (*p* = 0.14). However, this study did not describe dosing of supplementation and the patients who received IVIG still showed very low median levels after treatment, in comparison to known IgG target troughs which should be in the higher range, possibly implying undertreatment as one of the reasons for lack of noted effect. This study also did not look at the frequency of infections as an outcome measure. Lastly, the patients in this study were recipients of many different types of solid organ Tx and the majority had received multiple organ Tx, limiting the generalizability of this to pediatric kidney Tx recipients.

Based on current research, it remains unclear if simply the presence of hypogammaglobulinemia post-kidney Tx truly contributes to infections which impact outcomes of Tx. When reviewed closely, the majority of infections which were seen in the published studies were more suggestive of impairment of the innate or cellular immune components, rather than the adaptive antibody-mediated component. It could be hypothesized that hypogammaglobulinemia in many of these cases is actually a secondary finding due to the immunosuppression regimens which may be affecting both cellular and adaptive immunity, and that the infection risk is not predominantly IgG-related. It is also important to note here that Ig preparations contain not only IgG antibodies to bacterial antigens, but also antibodies to viral and fungal antigens. Additionally, there are other serum proteins (which regulate inflammatory pathways) which are present in the products which may contribute to immunity beyond the setting of IgG supplementation for low IgG levels [20]. Therefore, it is possible that any protective effects which have been conferred by IgG replacement may be due to the anti-viral (i.e., anti-CMV) or anti-inflammatory effects of IVIG rather than from replenishment of the antibody levels. Because of this, larger trials comparing patients receiving IVIG to those who are not being supplemented is important to document exactly what types of infections (identified by organism and location) are being modified in patients after Tx and whether low IgG level prior to initiation of supplementation makes a difference in effect. It is entirely possible that the beneficial effects of IgG product supplementation actually lie outside of the IgG replenishment (or are due to an unexpected antibody effect). Nevertheless, the awareness of the association of increased infections after Tx with Ig abnormalities is important since it may be a signal of overimmunosuppression and should prompt clinicians to more closely review and potentially modify the immunosuppressive regimen.

In summary, Ig abnormalities, especially severe hypo-IgG, appear to be associated with a higher risk and frequency of infections post-Tx, though the correlation may be secondary. These appear to be bacterial and fungal, with several studies also demonstrating an increased risk of CMV infections, although it is not clear that these would traditionally be infectious organisms that would be expected to be eradicated by antibody supplementation alone. Studies appear to demonstrate a link between the severity of IgG abnormalities and the risk of infections overall. Many infections documented were respiratory, though UTI, septicemia, and *C. difficile* gastroenteritis were also noted [21]. Mortality also appears to be increased in patients with Ig abnormalities, especially those with severe hypo-IgG.

Table 2 summarizes the types of major studies referenced above and their key findings.

**Table 2:** Key findings from the major published studies on the prevalence and implications of hypo-IgG in Tx recipients

First author	Reference number	Study design	Population demographics	Major findings
Broeders	[8]	Prospective observational	Adult kidney Tx	– Peak prevalence of hypo-IgG at 1–3 months (45%)
				– Small % with multiple Ig abnormalities
				– Higher early infection risk (mainly respiratory) in those with baseline low IgG and those with combined hypogammaglobulinemia at 3 months
Fernandez-Ruiz	[9]	Prospective observational	Adult kidney Tx	– Peak prevalence of hypo-IgG at 1 month (52%)
				– Low IgG at baseline (6.6%) associated with pre-Tx hemodialysis
				– Baseline low IgG and positive anti-HCV status were risk factors for post-Tx low IgG
				– Early and late hypo-IgG associated with several infections: pyelonephritis and bacterial, fungal and blood stream infections, and *C. difficile* enterocolitis
				– Correlation between severity of hypo-IgG and incidence + frequency of infections
Augusto	[10]	Prospective observational	Adult kidney Tx	– Largest adult study, but few patients with severe hypo-IgG
				– High incidence of hypo-IgG at day 15 (56%). Older age identified as risk factor for this
				– Later hypo-IgG predicted by early hypo-IgG and treatment for rejection
				– Sepsis between 6 and 12 months was the only infection associated with hypo-IgG
Madan	[11]	Prospective case control	Pediatric heart and kidney Tx	– Only prospective pediatric study (small patient numbers, 10 controls, 31 Tx patients, of whom 15 were kidney Tx recipients)
				– Mean IgG and IgM levels declined to below control levels from 1 month onward and remained lower
				– Hypo-IgG increased from 25% at 1 month to about 60% at 2 years
				– Link between Ig abnormalities and infections could not be ascertained due to study design and sample size limitations
Florescu	[12]	Meta-analysis	Various solid organ Tx (mostly adult patients)	– Overall rate of hypo-IgG in 1st year was 40% (kidney Tx recipients). Severe hypo-IgG in 8%
				– Overall rate of hypo-IgG lower in children (26%) compared to adults (data not restricted to kidney Tx recipients only)
				– 2.5 times higher odds of developing infections (respiratory, Aspergillus, CMV) in severe hypo-IgG group
				– 21.91 times higher odds of 1-year mortality in severe hypo-IgG group

## Hypogammaglobulinemia and bronchiectasis

In addition to the association between Ig abnormalities and post-Tx infections, data from small cross-sectional studies possibly suggest a concerning link between low Ig and the occurrence of bronchiectasis in patients following Tx, an otherwise uncommon pediatric condition. In the immune deficiency literature, hypogammaglobulinemia has been linked to bronchiectasis; as many as 30–60% of patients in some adult cohorts with antibody deficiency syndromes have been reported to develop this complication [22]. In a single-center retrospective study of MMF-treated Tx recipients, 2.4% developed bronchiectasis, all of whom were hypogammaglobulinemic and had experienced recurrent respiratory infections; notably, the Tx recipients who were not receiving MMF did not develop bronchiectasis [23]. Whether bronchiectasis in this population is causally related to low Ig directly, to the use of MMF, or as a consequence of the recurrent infections irrespective of Ig abnormalities, remains to be clarified. A link between MMF and bronchiectasis without hypogammaglobulinemia has also been shown, both in adults [24] and in children [25]; in some patients, discontinuation of MMF led to improvement in symptoms [24]. This direct link between MMF and bronchiectasis may be attributed to its deleterious effect on bronchial muco-ciliary clearance, as shown in an animal model [26]. Conversely, bronchiectasis has been noted in multicenter studies in patients who were not receiving MMF; in this large study of 46 patients with post-Tx bronchiectasis, 47% were hypogammaglobulinemic and 91% were receiving MMF at the time of diagnosis of bronchiectasis. Interestingly, in patients with primary immunodeficiency and bronchiectasis, some success has been achieved in markedly increasing the dosing of monthly IgG supplementation as a means of preventing breakthrough infections and limiting progression of further lung injury [27].

Suffice it to say that until further studies are done, recurrent respiratory infections in Tx recipients may warrant a relook at the maintenance immunosuppression patients are receiving, with a view to possibly substituting MMF with another agent, and an investigation into their Ig level.

## Link between Ig abnormalities and specific immunosuppressive agents

Published data, albeit limited, suggest that certain immunosuppressive agents or strategies may be associated with a higher incidence of Ig abnormalities after Tx. Some of these are briefly described below.

### Induction agents

#### High-dose (“pulse”) IV steroids

The parenteral use of steroid pulse therapy has been noted to be associated with a significant risk of developing severe hypogammaglobulinemia [28]. It is well known that large doses of glucocorticoids are lympholytic [29] and that corticosteroids may affect leukocyte migration and activation [30]. Furthermore, reduction of the naïve B cell compartment results in decreases in IgG level as well as the other Ig isotypes. In one study of 37 patients on high-dose steroids, up to 60% of the patients showed a marked reduction of IgG level while receiving treatment [31].

### Maintenance immunosuppression

#### MMF

MMF has all but replaced azathioprine (Aza) as a standard part of post-Tx maintenance immunosuppression [1]. Profound B cell depletion and hypogammaglobulinemia were reported in a pediatric kidney Tx recipient receiving maintenance therapy including MMF; the B cell and Ig abnormalities resolved following discontinuation of MMF, indicating a cause and effect relationship [32]. Mechanistically this can be explained by the inhibition by MMF of inosine monophosphate dehydrogenase, which is a key enzyme in the de novo pathway of purine synthesis in T and B cells [33]. Further evidence linking MMF to Ig abnormalities comes from a prospective clinical trial comparing Aza to MMF in 49 patients [34]. Patients received either Aza (n 17) or MMF (n 24). Ig levels were evaluated before and every month after Tx for 6 months. Unlike what was seen in the Aza group, there were significant decreases in IgG, IgM, and IgA levels in MMF-treated patients after 6 months; two patients (11.7%) in the Aza arm and 11 patients in the MMF arm (45.8%) developed at least one low level of Ig. The mean number of infection episodes was higher in the MMF group: recurrent UTIs developed in 8 patients, 7 of whom were receiving MMF. Seven of the 11 patients with low Ig levels in the MMF group had recurrent UTIs (63%), while no patient who had normal Ig levels developed recurrent UTIs. After 6 months, MMF was changed to Aza in these 7 patients; all but one normalized their Ig levels after 3 months of conversion and only two episodes of infection were recorded during this period.

### Rescue agents

#### Bortezomib

This proteasome inhibitor leads to apoptosis of plasma cells, which are the source of antibody production, and makes it a promising candidate in treating antibody-mediated rejection in patients after Tx. While studies have not been done in this population looking at hypogammaglobulinemia presumedly induced by loss of antibody-producing B cells, in studies of patients with systemic lupus erythematosus (most of whom had lupus nephritis), all patients had a decrease in IgG levels during their treatment [35]. Hypogammaglobulinemia (< 670 mg/dL) after bortezomib was identified in 5/12 (42%) patients, though the adverse events (mostly infections) were similar to patients who were not hypogammaglobulinemic.

#### Rituximab

Peripheral B cell depletion by this anti-CD20 monoclonal antibody is likely mediated through cell death via direct cytotoxicity, complement-dependent cytotoxicity, and antibody-dependent cellular cytotoxicity. While this is beneficial in the attempt to reduce antibody-mediated rejection, the innocent bystander effect of general hypogammaglobulinemia is also very prevalent in patients who receive rituximab. In one review of patients who received rituximab at a tertiary-care center for any reason, out of the 558 patients who had Ig levels monitored after treatment, 65% showed various levels of hypo-IgG (defined as < 600 mg/dL) [36]. Notably, the hypo-IgG and severe infections were more pronounced in those who had low IgG levels prior to treatment. In a review of ABO-incompatible kidney Tx recipients who had been treated with rituximab, an increase in infections was also noted, specifically increases in CMV infections, BK virus-associated nephropathy, and severe sepsis [37]. Other groups who did not necessarily find increased infections after rituximab treatment were notably using high-dose IgG supplementation for several months as part of their treatment protocol, precluding meaningful interpretation of their published data [38, 39].

## Conclusions and future directions

In summary, while Ig abnormalities are prevalent and associated with a higher risk and frequency of serious post-Tx infections (bacterial, viral, and fungal), a clear cause and effect relationship has yet to be established between these two factors. Based on data demonstrating a higher mortality risk in patients with hypogammaglobulinemia, and the availability of IVIG as a commonly used replacement therapy in patients with primary immunodeficiency, we would recommend that Ig levels be established in patients both pre- and post-Tx, to delineate those who might be more at risk during the first year after Tx. Strategies to address Ig abnormalities include adjusting maintenance immunosuppression (such as by substituting MMF with Aza) if feasible. Better documentation of the types of infections seen in patients with and without low IgG levels would be helpful to understand if there are effects which would be considered truly IgG-dependent. Finally, trials of compulsory IgG supplementation should be studied in patients post-Tx regardless of their IgG level, in order to establish if the infectious profiles which are seen more predominantly after kidney Tx (CMV, *E. coli*, *Enterococcus*) are actually modified by treatment in ways which would not be attributable to simply the correction of low IgG levels. Our hope is that greater attention will be directed towards this potentially treatable complication in children after kidney Tx and that more robust studies will be directed towards further exploration of this subject.

## Key summary points

1. Hypogammaglobulinemia is prevalent after kidney Tx and peaks around 1–3 months post-Tx
2. Ig abnormalities are associated with a high frequency of serious infections after Tx, in a level-dependent manner
3. Hypogammaglobulinemia may also be associated with a predilection for the development of bronchiectasis, resulting from recurrent respiratory infections
4. MMF, a very commonly used maintenance immunosuppressive agent, increases risk of Ig abnormalities which are reversible upon its discontinuation
5. There is a paucity of data on potential role of IVIG in patients post-Tx, both with and without hypogammaglobulinemia.

## Multiple choice questions (answers are given following the reference list)

1. Which of the following is an accurate statement pertaining to the epidemiology of hypogammaglobulinemia after kidney transplantation?
  A) Hypogammaglobulinemia is more common in children than adults after Tx
  B) The prevalence of hypogammaglobulinemia peaks at 12 months after Tx
  C) MMF is associated with a higher risk of Ig abnormalities compared to Aza
  D) Ig abnormalities are rare after Tx in the absence of an underlying primary immunodeficiency
2. Which of the following complications have been associated with post-Tx hypogammaglobulinemia**?**
  A) *Pneumocystis jirovecii* infections
  B) Secondary malignancies
  C) Bacterial sepsis
  D) Rotaviral gastroenteritis
3. Of the following immunosuppressive strategies, which has been shown to be useful in patients who develop post-Tx hypo-IgG and experience recurrent infections, while receiving an MMF-based immunosuppressive regimen?
  A) Substitute MMF with azathioprine
  B) Add an IL-2 receptor blocker to the regimen
  C) Replace MMF with methotrexate
  D) Start a short course of IV thymoglobulin
4. Which of the following statements pertaining to the treatment of hypogammaglobulinemia after Tx is true?
  A) Data support the use of IVIG replacement for all patients post-Tx with Ig abnormalities
  B) More frequent IVIG replacement therapy is required in post-Tx patients compared to those with primary immunodeficiencies
  C) IVIG therapy is contraindicated after kidney Tx
  D) There are no controlled trials assessing the role and indication for IVIG after kidney Tx
5. Which of the following statements is true about post-Tx bronchiectasis?
  A) The use of MMF has been linked to the development of bronchiectasis
  B) Data clearly support a causal link between Ig abnormalities and post-Tx bronchiectasis
  C) Post-Tx bronchiectasis is an indication for starting IVIG therapy
  D) The severity of hypogammaglobulinemia is a strong predictor for the development of bronchiectasis
